# Patterns of research utilization on patient care units

**DOI:** 10.1186/1748-5908-3-31

**Published:** 2008-06-02

**Authors:** Carole A Estabrooks, Shannon Scott, Janet E Squires, Bonnie Stevens, Linda O'Brien-Pallas, Judy Watt-Watson, Joanne Profetto-McGrath, Kathy McGilton, Karen Golden-Biddle, Janice Lander, Gail Donner, Geertje Boschma, Charles K Humphrey, Jack Williams

**Affiliations:** 1 Faculty of Nursing, University of Alberta, Edmonton, Canada; 2 Faculty of Nursing, University of Toronto and Hospital for Sick Children, Toronto, Canada; 3 Faculty of Nursing, University of Toronto, Toronto, Canada; 4 Toronto Rehabilitation Institute, Toronto, Canada; 5 School of Management, Boston University, Boston, USA; 6 Faculty of Nursing, University of British Columbia, Vancouver, Canada; 7 Data Library, University of Alberta, Edmonton, Canada; 8 Institute of Clinical Evaluative Sciences & Clinical Epidemiology and Health Services Research Program, Sunnybrook Health Sciences Centre, Toronto, Canada

## Abstract

**Background:**

Organizational context plays a central role in shaping the use of research by healthcare professionals. The largest group of professionals employed in healthcare organizations is nurses, putting them in a position to influence patient and system outcomes significantly. However, investigators have often limited their study on the determinants of research use to individual factors over organizational or contextual factors.

**Methods:**

The purpose of this study was to examine the determinants of research use among nurses working in acute care hospitals, with an emphasis on identifying contextual determinants of research use. A comparative ethnographic case study design was used to examine seven patient care units (two adult and five pediatric units) in four hospitals in two Canadian provinces (Ontario and Alberta). Data were collected over a six-month period by means of quantitative and qualitative approaches using an array of instruments and extensive fieldwork. The patient care unit was the unit of analysis. Drawing on the quantitative data and using correspondence analysis, relationships between various factors were mapped using the coefficient of variation.

**Results:**

Units with the highest mean research utilization scores clustered together on factors such as nurse critical thinking dispositions, unit culture (as measured by work creativity, work efficiency, questioning behavior, co-worker support, and the importance nurses place on access to continuing education), environmental complexity (as measured by changing patient acuity and re-sequencing of work), and nurses' attitudes towards research. Units with moderate research utilization clustered on organizational support, belief suspension, and intent to use research. Higher nursing workloads and lack of people support clustered more closely to units with the lowest research utilization scores.

**Conclusion:**

Modifiable characteristics of organizational context at the patient care unit level influences research utilization by nurses. These findings have implications for patient care unit structures and offer beginning direction for the development of interventions to enhance research use by nurses.

## Background

Investigators have described the difficulties and complexities of implementing change in practice [1], and increasingly we see calls for the design of more theory-informed interventions [2-4]. While calls to make nursing practice more research-based are common, research utilization investigators in nursing have argued that the use of research evidence is often not reflected in the delivery of nursing care despite the benefits of adopting research-based practices, and the increased availability of research to health professionals [5-7]. As a result, patients often do not receive optimal or effective nursing care. In response to this, we have seen accelerated efforts to develop interventions to increase the use of research in practice. However, relatively few reports exist about intervention studies in the area of research utilization for nurses, and those available have often not yielded positive results [8,9]. (One reason for this, we argue, is a failure to systematically account for the factors that influence nurses' use of research, or stated another way, to systematically account for the determinants of research utilization behaviour within the work context (*i.e*., organizational setting) of nurses.

Various individual, organizational, and most recently, contextual, factors have been argued as influencing the use of research by healthcare providers. Traditionally, the factors studied in nursing have tended to be determinants of research use that could be characterized as individual – such as age [10,11], attitude [11-13], clinical area [12,14], education [14-17], prior knowledge [15], employment status [10,16,17], experience [11,14,15], journals read [18,19], and recently, critical thinking dispositions [20]. In a systematic review of the literature on the individual determinants of research utilization by nurses, Estabrooks and colleagues identified a positive attitude toward research as both the most frequently studied individual determinant and the only one with a consistently positive effect [21]. Findings for all other individual determinants in that review were equivocal.

Less attention has been paid to the role of organizations and context in promoting research use [21-23]. Historically, a number of organizational factors thought to influence innovation adoption in industry and health services have been studied. Those shown to have an influence on innovation adoption include: organizational complexity [24], centralization [25], size [25,26], presence of a research champion [27,28], traditionalism [29,30], organizational slack [31], access to and amount of resources [19,29,32,33], constraints on time [34-36] and staffing [15,36], professional autonomy [35,37,38], geographic location (*i.e*., urban versus rural) [39], and organizational support [11,12,35,40,41].

Over the past decade, nurse investigators in the United Kingdom (UK) have called for more attention to contextual factors in promoting research use by healthcare providers [42-44]. They define context as 'the environment or setting in which the proposed change is to be implemented' and understand it to be comprised of three core dimensions: culture, leadership, and evaluation [42].

McCormack *et al*., in a concept analysis of context in relation to research implementation, define culture as the defining prevailing beliefs and values, consistency in values, and receptivity to change, among members of an organization or group [45]. Organizational culture, at least theoretically, affects clinician behaviors such as the adoption of research findings in practice. While positive effects of culture on research utilization have been suggested by several scholars in the field [42,46-49], to date, we have relatively little empirical evidence to support these assertions.

Leadership refers to the 'nature of human relationships' with effective leadership being proposed to give rise to clear roles, effective teamwork and effective organizational structures, as well as staff involvement in decision-making and approach to learning [45]. The effect of leadership has received much attention. Previous research has shown that leadership is instrumental for cultural change and has a strong effect on overall organizational performance [45,50,51]. There is also evidence that leadership is critical to nurses' decision-making processes [15,52]. and to creating a culture for evidence-based practice [6]. Additionally, research conducted in magnet hospitals in the United States (US) indicate that nurse leaders play a critical role in developing environments (*i.e*., contextual settings) that support nursing excellence and improved patient outcomes [53-55].

Evaluation, the third proposed core dimension of context, refers to feedback mechanisms (individual and system level), sources, and methods for evaluation [45]. Audit coupled with a feedback mechanism, where data is fed back to a unit's providers in the form of some kind of report, is one of the most commonly applied evaluation mechanisms used in healthcare to implement the adoption of research-based practices, and has been shown to have modest effects with physicians [56]. While its effect on nurses has been relatively untested, in one trial investigators reported that audit and feedback together with educational outreach and printed materials results in moderate improvements in nursing care [57], lending support to the importance of evaluation as a contextual predictor.

Additional support for investigating the role of context in research utilization comes from studies correlating specific contextual factors with research utilization behaviors of nurses. A number of investigators have correlated the impact of organizational structures, roles, and policies designed to promote research use with the actual use of specific research-based practices by nurses [13,14,26,58-60]. Studies examining the impact of context on research implementation in both the nursing [*e.g*., [52,61,62]] and organizational behaviour literature [*e.g*., [63]] also support the importance of contextual factors to research utilization, while stressing the interactivity among different contextual factors.

Despite growing support for the importance of organizational context to research utilization, little is known about which contextual factors are important for research utilization by nurses and how these factors operate. This lack of certainty was evident in the findings from a Cochrane systematic review [64] on organizational infrastructures for promoting research-based nursing interventions. The authors were not able to identify any studies meeting Cochrane standards.

A more recent review [65] that was not restricted to randomized control trials also assessed contextual factors and research utilization in nursing staff. These investigators reported that contextual factors (*e.g*., role, access to research, a favorable organizational climate towards research use, material support to attend conferences, time to read research, and organizational educational activities such as mini-courses) had statistically significant but inconsistent associations with research use. These findings suggest that while the contexts in which nurses work may be important to research use, further study in this area is needed.

Little consensus exists among researchers on the features that an 'ideal nursing unit' for research utilization would display. However, magnet hospital research in the US does give us some idea of what such an ideal unit would look like from staff retention and quality patient care perspectives. Consistently reported contextual and individual nurse characteristics of magnet hospitals include effective leadership (*i.e*., leaders who are visionary, enthusiastic, supportive, value education and professional development, maintain open lines of communication with staff nurses), the ability of staff nurses to establish and maintain therapeutic nurse-patient relationships, nurse autonomy and control, and collaborative nurse-physician relationships at the unit level [54,66,67]. The 'ideal nursing unit' for research utilization may exhibit similar individual and contextual characteristics, although this is yet to be empirically tested.

In summary, while an understanding of research utilization in nursing is growing, there are gaps in what is known about the factors that predict nurses' use of research. Knowledge of those factors would inform the development of interventions to increase the use of research in the service of improved patient care. Individual determinants of research use have been studied most frequently but findings are equivocal, making it difficult to plan interventions to facilitate research use, even at the individual level. Organizational determinants have been studied in industries beyond health; relatively few studies have been conducted in hospital settings or with nurses. Further, within healthcare organizations, nursing work is commonly organized at the patient care unit level, indicating a need to understand contextual factors at sub-levels (*i.e*., patient care units) within the organization. Few reports examine work at the patient care unit level. Before interventions to increase research use among nurses working in hospitals can be optimally designed, investigators need to identify and understand factors at both the hospital and the unit-levels [68]. In the study reported here, we focused at the patient care unit level.

### Purpose

The purpose of this study was to identify and examine individual and contextual factors at the unit level that influence research utilization among nurses working in acute care hospitals, and to identify any differences between adult and pediatric units. The specific purpose of the analyses reported in this paper was to conceptually model an ideal patient care unit, *i.e*., a patient care unit displaying features optimal for research use. We used a descriptive approach to develop an organizational archetype to examine determinants of research utilization at the patient care unit level. Using this approach, a framework for unit level research utilization was constructed based on our understanding of a model nursing environment.

### Theoretical framing

Rogers' diffusion of innovations theory [29,69]. has provided valuable insight into the field of research utilization. This theory explains the spread of new ideas using four main elements: the innovation, communication channels, time, and a social system. That is to say, diffusion is a process by which an innovation is communicated through certain channels over a period of time among members of a social system. It is not a single all-encompassing theory; rather it consists of four theoretical perspectives that relate to the overall concept of diffusion: innovation-decision process theory, the individual innovativeness theory, the rate of adoption theory, and the theory of perceived attributes.

While the study reported here does not represent an empirical test of the diffusion of innovation theory, we did use selected components of Rogers' [29] classical *Diffusion of Innovation *work (*i.e*., characteristics of the adopter and characteristics of the environment) to guide the selection of variables for the original survey [70] of which an abbreviated form was used in this study. For example, characteristics of the adopter included individual variables such as age and experience while characteristics of the environment included organizational and contextual variables such as unit culture and workload levels. See Additional File 1 for a complete listing of all variables included in the abbreviated version of the survey utilized in this study.

## Methods

### Design and Sample

Two adult surgical units (units one and two) and five pediatric surgical and specialty units (units three to seven) embedded in four metropolitan, tertiary level hospitals in two Canadian provinces, Alberta and Ontario, participated in the study. Ethical approval for the study was obtained from the Universities of Alberta and Toronto human research ethics committees and relevant university-affiliated institutional research ethics boards.

### Data Collection

Consistent with an ethnographic approach, both qualitative and quantitative data were collected. On each unit, fieldwork (participant observation, interviews, and focus groups) was conducted over a six-month period yielding qualitative data on nurses, physicians, other health professionals, patients and their families. Selected findings of the qualitative analysis have been reported elsewhere [71,72].

In months one and six of observations on each unit, two one-week periods of quantitative data collection occurred. Using survey instruments, data were collected on research use, organizational measures, critical thinking dispositions, unit workload, unit environmental complexity, and unit culture. The only inclusion criterion for participants was to be a registered nurse employed in one of the seven participating units. Sealed questionnaire packages were sent to all nurses working in the seven units, with two to three weeks allowed for completion. Participation was voluntary and anonymity was maintained. Posters, pamphlets, and informal communication with on-site data collectors during observation work were used as reminders to complete the questionnaires and return them to a centrally established location on the unit. Response rates varied with each instrument according to the time (*i.e*., month one or month six) of data collection (see Additional File 2). Across the seven units, 176 nurses participated at month one and 117 at month six. Analysis was performed on a sample of N = 235 [*i.e*. time one (N = 176) + time two (N = 117) minus nurses at time two who already filled out a survey at time one (N = 58)]. We excluded nurses at time two who already replied at time one so not to bias the findings by placing a greater weight on the responses from individuals responding twice. Due to the short time frame (six months) between times one and two, we also elected to combine responses from both periods. Further, our qualitative analyses during this six month interval did not show any evidence that the context of the units had changed and thus supported combining time one and time two responses. Table 1 provides the demographic profile of the nurses who participated in the study, and Table 2, a demographic profile of participating units.

**Table 1:** Demographic characteristics of participant nurses by unit (N = 235)

**Variables**		**Unit 1 **	**Unit 2 **	**Unit 3 **	**Unit 4 **	**Unit 5 **	**Unit 6 **	**Unit 7 **	**Overall **
		**(N = 37)**	**(N = 45)**	**(N = 15)**	**(N = 20)**	**(N = 19)**	**(N = 77)**	**(N = 22)**	**(N = 235)**
**Gender (%)**	Female	91.9	88.9	93.3	95.0	89.5	98.7	95.5	94.0
	Male	8.1	11.1	6.7	5.0	10.5	1.3	4.6	6.0
**Education (%)^a^**	LPN	14.3	0	13.3	10.0	5.3	0.0	0.0	4.3
	RN Diploma	57.1	44.2	66.7	80.0	47.4	39.0	40.9	48.9
	Bachelor's Degree	28.6	53.5	20.0	10.0	47.4	50.6	50.0	42.0
	Master's Degree	0.0	2.3	0.0	0.0	0.0	9.2	9.1	4.3
**Age (years)**	Mean (SD)	39.1 (10.6)	35.5 (8.8)	47.5 (9.3)	45.5 (7.6)	38.1 (9.6)	37.5 (8.4)	35.1 (7.8)	38.7 (9.5)
**Years in Nursing**	Mean (SD)	12.9 (9.8)	10.5 (9.1)	20.9 (8.6)	20.6 (8.4)	13.1 (7.9)	12.8 (8.9)	10.0 (8.1)	13.4 (9.4)
**Usual Shift Length (hours)**	Mean (SD)	10.6 (1.9)	11.6 (1.0)	11.1 (1.6)	8.0 (0.0)	11.4 (1.4)	11.8 (0.8)	11.2 (1.7)	11.1 (1.6)

^a^Numbers may not add up to 100% due to missing values.

SD = standard deviation

**Table 2:** Hospital (N = 4) and unit (N = 7) profile.

	**Unit Profile**
Unit 1	There were 37 RNs, including 17 full time and 14 part time RNs. The nurse manager was in charge of the unit. The majority of patients was older than 50 years and stayed on average 4–5 days.
(adult)	
Unit 2	There were 39 full time RNs, 17 part time RNs, and 10 casual RNs. The nurse manager was the leader on the unit. The patients stayed 1–3 weeks on average.
(adult)	
Unit 3 (pediatric)	Weekdays 4 nurses and 2 support staff worked the day shift. On nights and weekends, staff consisted of 2 nurses with support people. The clinical supervisor was the clinical leader on the unit; the unit manager took care of the managerial responsibilities for the unit.
Unit 4 (pediatric)	There were 17 full time RNs, 6 part time RNs, 2 LPNs and 11RNs relief in this unit. At the time of the study, the unit did not have a manager which was partly compensated for by the senior operating officer and the patient care director. The majority of the patients were discharged at that same day.
Unit 5 (pediatric)	Altogether there were 29 permanent nurses on this unit including 1 nurse educator and 2 LPNs. Local clinical leadership was provided by the clinical supervisor, while the unit manager performed the general administrative and leadership role, with some guidance from the senior operating officer. The average length of patient stay was 3 days.
Unit 6 (pediatric)	There was over 100 nursing staff in this unit, including 65 full time staff nurses, 25 part time staff nurses, 23 special assignment staff, 12 resource persons and 9 nurse specialists. The unit was administered by the unit manager working collaboratively with the medical clinical directors and the child health services manager.
Unit 7 (pediatric)	There was 37 nursing staff including the unit manager and the child health services manager. The average daily admissions were 4–5.

The seven pediatric and adult acute care units were embedded in four urban, tertiary level hospitals in two cities, each affiliated with a university. Of the four hospitals: one was a dedicated pediatric center, one had adult and pediatric units, and two were dedicated adult care hospitals. The seven units included five pediatric units and two adult surgical units.

### Instruments

Six instruments were used to collect the quantitative data: A Demographic (DEM) Inventory, a Research Utilization Survey, the Environmental Complexity Scale (ECS), the Nursing Unit Cultural Assessment Tool (NUCAT) Version 3, the Project Research in Nursing (PRN) 80, and the California Critical Thinking Disposition Inventory (CCTDI). These are described briefly in sections that follow. The ECS and PRN were both completed by research associates on the unit during the two separate week-long quantitative data collection periods, while the remainder of the instruments were self-administered by the nurses. A sample of the items and scales used to measure the study variables and corresponding reliability coefficients for scales are shown in Additional File 1.

### Demographic (DEM) inventory

The DEM developed for this study, included questions on gender, age, education, hours of work per week, length of shift, years working in nursing, membership in nursing organizations or groups, and the number of years worked on the unit.

### Research utilization survey

The Research Utilization Survey was first developed and reported by Estabrooks [70,73]. A shortened version of the original research utilization survey was used in this study. The shortened version consisted of 22 questions divided into three sections: research utilization, kinds and sources of knowledge for practice, and organizational characteristics.

### Environmental complexity scale (ECS)

The ECS [74-76] was designed to assess the amount and degree of work disruption experienced by nurses over the course of a shift. Since its original publication in 1997, the scale has undergone several pilot tests, reviews, and modifications. The version used in this study consisted of 23 items divided into three subscales: unanticipated changes in patient acuity, re-sequencing planned in nursing work to accommodate others, and influence of students. Individual items on each subscale were coded 0–10 (high increase to high decrease) and summated to obtain final subscale scores.

### Nursing unit cultural assessment tool v3 (NUCAT3)

The NUCAT3 was developed by Coeling [77,78]. The primary purpose of this tool is to describe and understand nurses' immediate work group in a unit setting. A list of 50 items in the form of questions, representing various behaviours is listed mid-page in the questionnaire. A five-point scale on the left and right of each item allows nurses to indicate how important the behaviour is to them personally (left) and to the group as a whole (right). Based on the responses to the 50 items, five subscales were conceptually created to reflect specific cultural indicators reflective of the behaviours for the nurses in this study. These subscales were co-worker support, questioning behaviour, continuing education, work values-creativity, and work values-efficiency.

### Project research in nursing 80 workload measurement tool (PRN)

The PRN is a Canadian classification system used to measure the level of nursing care required by patients in hospitals and nursing homes [79]. It consists of seven major categories: respiration, feeding and hydration, elimination, hygiene and comfort, communication, treatments, and diagnostic procedures. Each category provides a list of patient related needs, which are assigned a point value based on frequency and complexity. The total score, determined by summing up the points from each of the seven categories, is multiplied by five minutes to determine the direct care time estimate for each patient. The higher the point value the greater the amount of direct care required. The PRN method of measuring care required has been tested extensively and has undergone several iterations since its development in 1972. In 1978, Chagnon, Audette, Lebrun, and Tilquin reported its construct and predictive validity [80].

### Critical thinking dispositions inventory (CCTDI)

The CCTDI is a 75-question, six-point 'agree/disagree' Likert-type scale. There are seven subscales to the inventory: truth-seeking, open-mindedness, inquisitiveness, systematicity, maturity, self-confidence, and analyticity. The maximum overall score attainable on this tool is 420, with each subscale contributing a maximum of 60 points. The standard scores for each subscale and all scales combined are 40 and 280 respectively. A score less than 40 on any subscale or less than 280 overall indicates limitations or weakness, whereas subscale scores of 50 or higher and overall scores at 350 or higher indicate a strength in critical thinking dispositions [81].

### Analysis

While research utilization and possible explanatory variables were measured at the individual level, the unit of analysis in this study was the patient care unit. To create unit scores, data collected at the level of the individual nurse were aggregated to the level of the patient care unit by calculating group means. When Cronbach alpha was assessed, this was done at the individual level. One-way analysis of variance (ANOVA) was performed for each variable using the unit as the group variable. The source table from the one-way ANOVA was used to calculate the following indices: 1) interclass correlation ICC (1) = (BMS - WMS)/(BMS + [K - 1] WMS), where BMS is the between-group mean square, WMS is the within-group mean square, and K is the number of subjects per group. The average K for unequal group size was calculated as K = (1/[N - 1]) (ΣK - [ΣK^2^/ΣK]); 2) interclass correlation ICC (2) = (BMS - WMS)/BMS; 3) η^2 ^= SSB/SST, where SSB is the sum of squares between groups and SST is the sum of squares total; and 4) ω^2 ^= (SSB - [N - 1]WMS)/(SST + WMS). For each nursing characteristic analyzed, there was strong agreement among nurses in each given unit when ICC(1) was greater than 0.1. Aggregated data were considered reliable when the *F *statistic from the ANOVA table was statistically significant (*p *< 0.05) and/or ICC(2) was greater than 0.60 [82]. An indicator of effect size was η^2^, which was the proportion of variance in the individual factor accounted for by group membership [83], and ω^2 ^was a measure of the relative strength of the aggregated variable at the group level [84]. Table 3 contains the reliability and validity values of the data aggregated at the unit level. Both η^2 ^and ω^2 ^are measures of validity of the aggregated data at the patient care unit level.

**Table 3:** Reliability and validity of data aggregated at the unit level

**Variable**	**ANOVA**	**Degrees of Freedom**	**ICC(1)**	**ICC(2)**	**η^2^**	**ω^2^**	**Alpha**
Overall RU	5.83**	6,264	0.11	0.83	0.12	0.00	--
Authority	2.85*	6,303	0.04	0.65	0.05	0.00	--
Attitude	1.08	6,303	0.00	0.07	0.02	0.00	--
Intent	2.34*	6,298	0.03	0.57	0.05	0.00	--
Belief	2.43*	6,285	0.03	0.59	0.05	0.00	0.85
People support	4.60**	6,181	0.09	0.78	0.14	0.00	0.89
Organizational support	21.56**	6,204	0.34	0.95	0.40	0.28	0.85
Re-sequencing	12.21**	6,359	0.19	0.92	0.17	0.06	0.81
Students	1.57	6,133	0.02	0.36	0.07	0.00	0.75
Acuity	16.15**	6,364	0.24	0.94	0.21	0.11	0.84
Coworker support	2.36*	6,149	0.06	0.58	0.09	0.03	0.72
Education	1.46	6,144	0.02	0.32	0.06	0.00	0.64
Behavior	1.62	6,152	0.03	0.38	0.06	0.00	--
Creativity	0.86	6,155	0.01	0.11	0.03	0.00	--
Efficiency	0.92	6,154	0.01	0.12	0.04	0.00	--
Total PRN	260.32**	6,1334	0.59	1.00	0.54	0.48	--
Total CT	1.54	6,140	0.03	0.36	0.06	0.00	--

(a) Analysis of variance (ANOVA): Measure used to compare differences in mean scores across seven units;

(b) p value for ANOVA F-statistics:* p < .05; **p < .01. The denominator, degree of freedom, differs for some variables owing to different instruments;

(c) ICC = interclass correlation;

(d) η^2^: proportion of total information in a given factor at the individual level, which is captured by aggregated data;

(e) ω^2^: provides a relative measure of the strength of an independent variable, small effect < 0.06; medium effect, 0.06–0.15; large effect > 0.15

To index diversity across units, a coefficient of variation was computed and used in a correspondence analysis. A coefficient of variation is a quotient of standard deviation over the mean, and allows distributions among different units to be compared [85]. It is expressed as a percentage, which constitutes a relative measure of dispersion. In order to assess the relationship between various factors across the seven units, the coefficient of variation was computed and the resulting quotient was multiplied by 100 and denoted in the variation index. Variation indices are commonly used in research for making comparisons [86-88]. In this study, the variation index matrix was then analyzed using correspondence analysis, which is a statistical visualization method for picturing the associations among the variables of a two-way contingency table. The object of a correspondence analysis is to obtain a graphical display in the form of a spatial map of rows (units) and columns (factors), not only with respect to their marginal profile, but also among each other. Here, we used correspondence analysis to explore the association between the pattern of factors (or determinants) and units. It should be noted however that correspondence analysis is an exploratory technique, based on a philosophical orientation that emphasizes the development of models that fit the data, rather than the rejection of hypotheses based on the lack of fit (Benzecri's 'second principle'). Therefore, statistical significance tests are not customarily applied to the results of a correspondence analysis, and are not needed for the clustering of factors produced in a correspondence analysis [89,90].

## Results

### Reliability of aggregated nursing measures

The reliability properties of the aggregated nursing data at the unit level are shown in Table 3. These properties supported the reliability of the aggregated data at the unit level for over half of the variables: overall research utilization, authority, intent, belief, people support, organizational support, re-sequencing, acuity, co-worker support, and total PRN. Statistically significant (*p *< .05) *F *statistics and/or ICC(2) values greater than 0.60 indicate greater reliability and justification for aggregating the variables at the unit level. The ICC(1) values greater than 0.00 indicate some degree of perceptual agreement of nurses about the mean values within each unit. That is, the nurses' perceptions about their own unit were highly similar. However, the relative effect sizes for both η^2^and ω^2 ^values were smaller, with η^2 ^indices ranging from 0.02 to 0.54 and ω^2 ^indices ranging from 0.00 to 0.48. Negative ω^2 ^indices are reported as 0.00 [84,91]. The smaller η^2^and ω^2 ^indices suggest that, as we aggregated data, our ability to assign the same meaning for a variable at the unit level that we had at the individual level lessened considerably.

### Research utilization

**Figure 1:**
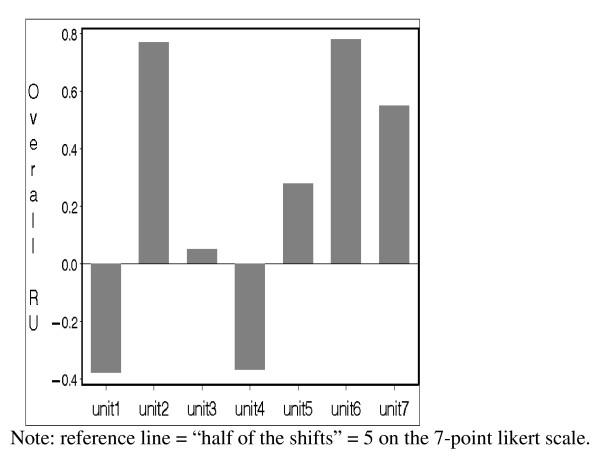
**Research utilization scores by unit**. Note: reference line = "half of the shifts" = 5 on the 7-point likert scale.

Adjusted overall research utilization scores were used. Overall research utilization was assessed with a single question asked at three different points in the questionnaire: 'Overall, in the past year, how often have you used research in some aspect of your nursing practice?' Repeated measures analysis of variance revealed that the overall research utilization scores increased significantly from the first to the second question (*p *< 0.001), and from the second to the third question (*p *< 0.05). Adjusted overall research utilization scores were obtained by taking a weighted average of the score obtained from the three times. The first inquiry was given a weight of 1/6, the second was given a weight of 2/6, and the third was given a weight of 3/6. We assigned higher weights to the research utilization question each time it appeared in the questionnaire because participants learned more about research utilization over the course of questionnaire completion. We reasoned that their answers were more reflective of their true scores each time they encountered the question, thus requiring a greater weight be placed on later inquiries. Figure 1 shows the adjusted overall research utilization scores with 'used research on about half the shifts' (five on the seven-point scale) as a reference line across the seven units. Analysis of variance indicated that statistically significant differences existed among units on the overall research utilization score (*p *< 0.001).

As illustrated in Figure 1, the seven units fell into three main groupings with respect to research utilization which we categorized as low (units one and four), moderate (units three and five), and high (units two, six, and seven) research utilization units. Units seven (pediatric), two (adult) and six (pediatric) had the highest mean scores of research utilization with means of 5.55 (SD = 1.31), 5.77 (SD = 1.22) and 5.78 (SD = 1.10) respectively. We found no statistically significant difference between units two, six, and seven however on research utilization scores (ANOVA, *p *> 0.05). In contrast, units one (adult) and four (pediatric) were the only units with mean scores of research utilization less than 5. Again, there was also no statistically significant difference between units one and four on research utilization scores (ANOVA, *p *> 0.05).

### Factors influencing research utilization

Table 4 displays the mean scores of selected variables from the Research Utilization Survey, Environmental Complexity Scale (ECS), Nursing Unit Cultural Assessment Tool v3 (NUCAT3), as well as total scores for the Project Research in Nursing (PRN) 80, and the Critical Thinking Dispositions Inventory (CCTDI).

**Table 4:** Mean scores and standard deviations by unit

	**Unit 1 (Adult)**	**Unit 2 (Adult)**	**Unit 3 (Pediatric)**	**Unit 4 (Pediatric)**	**Unit 5 (Pediatric)**	**Unit 6 (Pediatric)**	**Unit 7 (Pediatric)**	**Overall**
**Research Utilization Survey**								

People Support (Max score = 30)	17.94 (7.05)	20.70 (6.51)	18.79 (6.32)	16.44 (7.97)	20.29 (7.87)	21.18 (6.47)	20.30 (6.31)	19.94 (6.87)
Autonomy/Authority (Range is 0–4)	2.52 (0.81)	2.86 (0.95)	3.11 (0.81)	2.53 (1.01)	2.96 (0.74)	2.59 (0.82)	2.96 (0.74)	2.72 (0.86)
Attitude (Range is 0–4)	2.91 (0.92)	3.19 (0.83)	3.00 (0.75)	2.72 (0.96)	2.92 (0.95)	3.02 (0.82)	2.93 (0.92)	3.00 (0.87)
Intent (Range is 0–2)	1.78 (0.42)	1.76 (0.43)	1.53 (0.51)	1.44 (0.51)	1.52 (0.51)	1.67 (0.49)	1.70 (0.47)	1.68 (0.48)
Belief Suspension (Range is 0–4)	2.13 (0.99)	2.37 (0.95)	2.47 (1.15)	2.29 (1.13)	2.37 (1.13)	2.50 (0.87)	2.11 (0.87)	2.34 (0.97)
Organizational Support (Max. Score = 25)	11.70 (4.23)	13.61 (5.15)	11.94 (5.32)	7.89 (2.65)	11.13 (2.85)	15.28 (4.14)	14.89 (2.36)	13.30 (4.61)
Overall Research Utilization #1	3.94 (1.78)	5.43 (1.50)	4.47 (1.99)	3.59 (1.54)	4.43 (1.99)	5.18 (1.61)	5.16 (4.41)	4.80 (1.75)
Overall Research Utilization #2	4.67 (1.85)	5.51 (1.61)	5.21 (1.89)	4.12 (1.87)	5.24 (1.81)	5.69 (1.39)	5.59 (1.60)	5.30 (1.68)
Overall Research Utilization #3	4.83 (1.91)	5.83 (1.25)	5.06 (1.82)	5.19 (1.72)	5.56 (1.78)	5.93 (1.30)	5.59 (1.42)	5.56 (1.57)
Adjusted (weighted) Overall research Utilization Score	4.62 (1.62)	5.77 (1.22)	5.05 (1.82)	4.63 (1.34)	5.28 (1.63)	5.78 (1.10)	5.55 (1.31)	5.24 (1.43)

**Environmental Complexity Scale**								

Re-sequencing of work (Range is 0–50)	28.50 (9.66)	35.39 (7.96)	28.24 (6.53)	30.0 (8.98)	24.78 (5.75)	27.21 (6.03)	30.72 (7.94)	29.45 (7.94)
Influence of Students (Range is 0–20)	14.33 (5.30)	12.18 (1.78)	11.37 (3.13)	10.00 (0.00)	10.91 (2.79)	12.61 (3.72)	11.00 (2.61)	11.77 (3.35)
Changing patient acuity (Range is 0–90)	54.77 (18.68)	67.30 (11.93)	48.01 (9.95)	52.35 (13.70)	50.70 (10.53)	53.27 (12.27)	57.05 (11.74)	55.76 (13.72)

**Nursing Unit Cultural Assessment Tool (Group's Behavior)**								

Co-worker support (Range is 0–10)	7.56 (2.20)	8.42 (1.69)	7.83 (1.75)	8.00 (1.25)	9.00 (1.07)	7.15 (1.74)	7.71 (1.49)	7.78 (1.78)
Questioning behavior (Range is 0–5)	4.04 (0.82)	4.21 (0.83)	4.58 (0.52)	4.36 (0.67)	4.47 (0.64)	4.23 (0.84)	3.83 (0.92)	4.21 (0.81)
Continuing education (Range is 0–20)	14.39 (2.74)	15.65 (2.98)	14.83 (2.67)	14.44 (2.60)	15.73 (2.21)	15.96 (2.27)	14.94 (2.07)	15.32 (2.52)
Work values (creativity) (Range is 0–5)	3.62 (0.98)	3.96 (0.89)	3.58 (0.79)	3.27 (0.79)	3.93 (0.70)	3.60 (0.92)	3.53 (0.91)	3.66 (0.89)
Work values (efficiency) (Range is 0–5)	4.31 (0.84)	4.36 (1.00)	4.08 (1.00)	4.10 (0.74)	4.13 (0.35)	4.24 (0.72)	3.78 (0.94)	4.19 (0.82)

**Project Research in Nursing 80**								

Total PRN	255.42 (108.15)	248.37 (82.98)	188.54 (81.70)	149.69 (24.59)	217.41 (83.17)	592.04 (157.84)	307.94 (124.86)	303.84 (184.41)

**Critical Thinking Dispositions Inventory**								

Total CCTDI (Max score = 420)	286.26 (28.39)	281.65 (31.38)	256.71 (15.96)	283.86 (25.63)	288.60 (25.57)	279.61 (25.54)	291.00 (29.33)	281.78 (27.58)

#### Research Utilization Survey

With respect to the Research Utilization Survey, unit six (pediatric) had the highest aggregated mean scores for three of the six subscales: people support, belief suspension, and organizational support. In contrast, unit four (pediatric) had the lowest aggregated mean scores for four of the six subscales: people support, attitude, intent, and organizational support. Comparisons of research utilization measures showed that adult and pediatric units did not differ significantly.

#### Environmental complexity scale (ECS)

There are three subscales on the ECS: re-sequencing of work, influence of students, and changing patient acuity. Statistically significant differences were noted between the seven units on the three subscales (re-sequencing of work – ANOVA F-test statistic = 13.352, *p *< 0.001; influence of students – ANOVA F-test statistic = 2.615, *p *= 0.020, changing patient acuity – ANOVA F-test statistic = 16.575, *p *< 0.001). Generally speaking, adult units scored higher than pediatric units (see Table 4). The overall mean score for re-sequencing of work was 29.45 (SD = 7.94). Unit two (adult) scored the highest (mean = 35.39, SD = 7.96) and unit five (pediatric) scored the lowest (mean = 24.78, SD = 5.75). The overall mean score for influence of students was 11.77 (SD = 3.35). Unit one (adult) scored the highest (mean = 14.33, SD = 5.30) and unit four (pediatric) scored the lowest (mean = 10.00, SD = 0.00). The overall mean score for changing patient acuity was 55.76 (SD = 13.72). Unit two (adult) scored the highest (mean = 67.30, SD = 11.93) and unit three (pediatric) scored the lowest (mean = 48.01, SD = 9.95).

#### Unit culture

The NUCAT3 assesses and describes unit culture on five subscales: co-worker support, questioning behavior, continuing education, work values – creativity, and work values – efficiency. Units two (adult) and six (pediatric) had the highest aggregated mean scores on three of these dimensions of group behavior: work values – creativity, work values -efficiency, and continuing education. Units three (pediatric) and five (pediatric) had the highest aggregated mean scores on questioning behavior and co-worker support respectively. Differences between adult and pediatric units were not noted to be statistically significant.

#### Workload

The overall PRN aggregated mean score for each unit ranged from 149.69 (unit four – pediatric) to 592.04 (unit six – pediatric). Statistically significant differences between adult and pediatric units were noted for the total score (*p *< 0.001).

#### Critical thinking

The overall aggregated mean scores of critical thinking dispositions (CCTDI) for the seven units ranged from 256.71 (unit three – pediatric) to 291.00 (unit seven – pediatric). Comparisons of critical thinking dispositions showed that adult and pediatric units did not differ significantly with respect to overall aggregated mean critical thinking scores.

### Correspondence analysis

**Figure 2:**
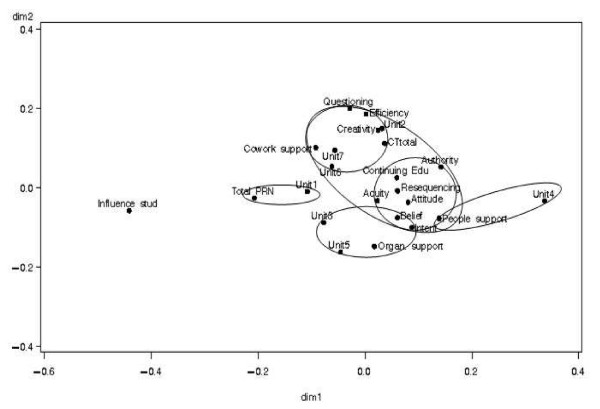
Overall correspondence analysis map illustrating unit clustering with contextual factors.

The full set of variables (except individual nurse demographic variables) was entered into a correspondence analysis, revealing a space (see Figure 2) structured along two dimensions, which captured two thirds of the variability (65.99%). As illustrated in Figure 2, critical thinking dispositions and unit culture (as measured by work values – creativity, work values – efficiency and questioning behavior) were found to be close to unit two (adult), a high research utilization score unit with a research utilization mean of 5.77, indicating an association between these factors and this unit. Unit culture (as measured by co-worker support) appeared to have a close relationship with units six (pediatric) and seven (pediatric), also high research utilization units. Another cluster included authority to use research, unit culture (as measured by importance of access to continuing education), environmental complexity (as measured by work re-sequencing, changing patient acuity), attitude toward research, people support, belief suspension, and intent to use research, suggesting this cluster of factors are consistently associated with each other. An additional factor, influence of students, was far from all of the other factors, reflecting dissimilarity with the other factors across the seven units. Unit four (pediatric) was also far from other units, but close to the factor of people support. We also observed that nursing workload (*i.e*., total PRN score) was more associated with unit one (adult), and organizational support with unit five (pediatric).

### Superimposing the research utilization scores onto the correspondence analysis map

Superimposing findings from the research utilization scores onto the correspondence map revealed interesting results. Using the results from the overall research utilization scores, the units cluster in three distinct groups: low (units one and four), medium (units three and five) and high (units two, six, and seven). These are summarized in Table 5.

**Table 5:** Mapping of correspondence analysis results onto unit groups based on research utilization scores

**FACTORS**	**Low Group**	**Medium Group**	**High Group**
	*Units 1 and 4*	*Units 3 and 5*	*Units 2, 6, and 7*

Influence of students (ECS)	X		
People support (RU)	X		
Total PRN score (PRN80)	X		
Organizational support (RU)		X	
Belief suspension (RU)		X	
Intent (RU)		X	
Changing patient acuity (ECS)			X
Re-sequencing of work (ECS)			X
Attitude (RU)			X
Continuing education (NUCAT3)			X
Critical thinking (CCTDI)			X
Work values: Creativity (NUCAT3)			X
Work values: Efficiency (NUCAT3)			X
Authority (RU)			X
Questioning behavior (NUCAT3)			X
Coworker support (NUCAT3)			X

The three groupings (low, medium, high) were based on the aggregated research utilization scores for each unit

'X' means that the factor sat closest to the respective unit group

When the research utilization scores in the high group (adult unit two, pediatric units six and seven) are superimposed onto the correspondence analysis map they appear close to one another in physical proximity (see Figure 2) suggesting they share similar characteristics. However units six and seven were closer to each other than to unit two indicating there may be subtle differences between factors that determine research use in adult compared to pediatric units. The following factors clustered around the three high research utilization units: changing patient acuity, re-sequencing of work, attitude toward research, critical thinking dispositions, importance of access to continuing education, work values (creativity and efficiency), authority, questioning behavior, and co-worker support, indicating an association between high research utilization units and these factors. Some of these factors clustered more closely around the units than others indicating a possible stronger relationship with research use: unit culture [as measured by work values (creativity and efficiency), authority, questioning behavior], and critical thinking dispositions.

After superimposing the research utilization scores onto the correspondence analysis map we also realized that the units in the low group (units one and four) were unlike the other units. Units one and four had the lowest levels of overall research utilization scores and subsequently plotted farther away from the other units (and each other) in the correspondence analysis map. Nursing workload (*i.e*., total PRN) and people support clustered close to unit one and unit four, respectively, indicating these two factors may be associated with lower research utilization units.

When units in the medium research utilization group (units three – pediatric and five – pediatric) were superimposed onto the correspondence analysis plot we discovered a third clustering. In particular, we saw that these units are not like the units in the two other groups. Organizational support, belief suspension, and intent to use research clustered more proximally to the medium group than the other two cluster patterns, indicating an association between units with moderate research utilization and these three factors.

## Discussion

This discussion focuses on individual and contextual factors and their role in research utilization by nurses. This study was exploratory in nature. Data were collected from nurses employed on seven units. The unit of analysis was the patient care unit and our sample size was thus seven. Findings and interpretations must therefore be interpreted cautiously and premature generalizations avoided.

Other research utilization investigators have explored several of the factors that we studied in this project. In particular, links between research utilization and attitudes toward and beliefs about research [11,60,92], continuing education [10,19,93], critical thinking [20], and support for research use [11,40,60] at the individual nurse level have been previously investigated. However, our unit of analysis was the patient care unit, and therefore, the comparisons described between the findings of this study and past research where the individual nurse was the unit of analysis should be interpreted with caution.

Some of our findings are consistent with previous work in the field. For instance, our finding that patient care units with high and moderate levels of research use had the highest levels of co-worker and organizational support respectively is not new. Champion and Leach [11] found support from the unit director, chairperson, and director of nursing to be positively correlated with nurses' use of research in their practice nearly 20 years ago. Hatcher and Tranmer [40] also reported small positive significant associations between the amount of organizational support nurses perceived and their use of research in practice. In addition, Varcoe and Hilton [60] demonstrated that the use of specific research-based practices was correlated with organizational support.

Our finding that patient care units with the highest levels of research utilization had, on aggregate, nurses with more positive attitudes about research use is also not new. Nurses' positive attitude towards research has been consistently shown to be associated at statistically significant levels with research use [21].

Authority to use research was also associated with higher levels of research utilization. While there is no literature that directly associates authority and research utilization, there is support for this concept in the 'barriers to research utilization' literature in nursing. Several investigators have noted that one of the most consistently reported barriers to using research in practice for nurses is 'lack the authority to implement change based on research findings' [94-102].

Our findings run counter to the work of some investigators. For example, Profetto-McGrath *et al*. [20] reported a statistically significant positive correlation between critical thinking dispositions and research utilization. Given the work by Profetto-McGrath and colleagues, we would expect to see high critical thinking dispositions scores for nurses on units two, six, and seven (*i.e*., high research utilization units) in comparison to the nurses on the other study units. However, there were no statistically significant differences between nurses on total critical thinking dispositions scores even though critical thinking dispositions did cluster around the 'high' research utilization units in the correspondence analysis. The Profetto-McGrath, *et al*. work was conducted on a subset of the data used for this study. However, their unit of analysis was at the level of the individual nurse, possibly accounting for differences. It may be that critical thinking dispositions are most productively studied as an individual level phenomenon, as suggested by our non-significant ANOVA F-statistic and ICC(2) of < 0.60 for the total critical thinking dispositions aggregated mean scores (see Table 3).

The culture of a unit defines the behavior of nurses through observable artifacts, values (*i.e*., norms, social principles and ideologies), beliefs, and attitudes [46,103]. As such, it constitutes a potential contextual determinant of research utilization. In this study, 'high' research utilization units had the highest aggregated mean unit culture scores (as measured by importance of access to continuing education, work values – creativity, work values – efficiency, questioning behavior, and co-worker support) indicating that variables associated with unit culture reflect the vitality with which research utilization can be promoted within patient care units. Positive effects of culture on research utilization have been suggested by several scholars in the field [42,46,47] but, to date, we have relatively little empirical evidence to support these assertions. For example, while several previous studies have examined continuing education, an element of unit culture as measured by the NUCAT3, in relation to nurse research utilization behavior, findings have been equivocal. McCleary and Brown [104] found taking a course about research design was positively associated with research utilization. Rodgers [36] found that the number of study days attended was associated with using more research in practice. However, other investigators, have not found similar associations [10,36,105]. Further research examining the link between nurse research utilization and continuing education will be necessary before a more definitive statement on its value as an intervention to increase research use in practice can be made. In addition to continuing education, recent work by Belkhodja *et al*. [48] found specific aspects of unit culture, such as the unit's research culture (*i.e*., research as the preferred source of information) and the intensity of use of research sources by the unit's members to also be positively correlated (*p *< 0.05) with research utilization by healthcare professionals on hospital units.

Pepler *et al*. [49] in a multiple case-study of research utilization on eight acute care units also found unit culture to be a principal factor linked to patterns of research utilization. However, while Pepler and colleagues identified several aspects of unit culture that were important to research utilization (*e.g*., harmony of research perspectives, motivation to learn, goal orientation, creativity, critical inquiry, mutual respect, and maximization of resources) they also reported that the components of unit culture were tightly intertwined resulting in a complexity which represents a distinctive culture for each unit. While this represents early support for unit culture as a factor in research utilization behaviours of nurses, further empirical support is needed before a statement regarding the association between unit culture and research use can be confidently made.

In addition to the factors discussed above, we reported a number of other factors that have not been previously studied with respect to nurses' research utilization behavior. For example, links between research utilization and nursing workload, patient acuity, and re-sequencing of work have not been previously explored, suggesting fruitful new avenues of inquiry. While we located no reports of these concepts having been studied in relation to research utilization, there are many studies reporting on nurse perceived barriers to using research. Among these, investigators consistently report a lack of time to read research and implement findings as one of the most frequently identified barriers [37,97,99]. Little clarification of what is meant by time has been offered in these studies, although an implicit assumption is that nurses' lack of time prevents research use. Our findings suggest this may not be the case. Two of the units with the highest workloads in the study reported here were units one and two (both adult units). Unit one was classified as a 'low' research utilization unit and unit two, a 'high' research utilization unit, making it difficult to ascertain the direction of the relationship between workload and research use. However, these findings do lead us to propose that there may be contextual differences between units (*e.g*., primary versus team nursing models, patient case mix, patient care acuity, healthcare team composition) that influence nurses' research use.

In addition to the unit contextual and individual factors identified in the correspondence analysis as important to research use, the 'high' research utilization units (*i.e*., units two, six, and seven) also had the highest proportions of baccalaureate and master prepared nurses and the youngest nurses (see Table 1). Education and age have been investigated in numerous previous research utilization studies and investigators have reported equivocal effects, at best, on nurse research utilization behaviours. For example, several studies showed no statistically significant association between education and research use [60,92,106]. while others showed the use of research in practice to be higher among nurses with baccalaureate/masters degrees compared to those with registered nurse diplomas [10,36,105]. Similarly, age has not been demonstrated to predict research use [10,19,92]. For this reason, and because we were interested in identifying modifiable, or at least more readily modifiable, factors influencing research use, we chose not to enter age and education into our correspondence analysis. Other individual characteristics such as questioning behaviours and belief suspension were entered in the correspondence analysis because we postulated they would be modifiable through continuing education. Age is not modifiable and education, while modifiable, would require long-term commitment.

### The archetypical unit

The specific purpose of the analyses presented in this paper was to model an ideal patient care unit. In such an ideal or archetypical patient care unit factors would be optimized to facilitate research use. We identified a number of such modifiable factors or characteristics that were associated in this study with patient care units that reported greater research use (see Table 5). In such units, these characteristics included unit culture, (specifically: co-worker support, questioning behavior, importance of access to continuing education, work values – creativity, work values – efficiency), environmental complexity, workload, authority to use research, positive attitudes towards research, and stronger critical thinking dispositions. These findings illustrate both the complex nature of research utilization and the shortcomings of models that address only individual or unit level dimensions. Either of these dimensions (individual, unit/contextual), while necessary, is insufficient to adequately explain the complex behavior changes required by nurses who use research optimally and appropriately. Importantly, our modeling of such an archetypical patient care unit, allowed us to identify contextual factors (*e.g*., importance of access to continuing education, co-worker support, questioning behavior) that can be modified to increase research use.

### Study Limitations

While this was a multi-centre study, the sample size in the analyses reported here was relatively small and may have been inadequate to detect differences between units for some of the variables. This study was also exploratory in nature and the findings drawn from seven units and the nurses employed on those units. The results must be interpreted with caution and are not generalizable either to nurses or units. While this study sheds light on the factors that may influence research use at the patient care unit level, further research is needed to expand on this knowledge. In particular, contextual factors (nursing workload, patient acuity, and re-sequencing of work) that have not been previously reported in relation to research use suggest directions for study.

While we were able to identify and build a model of an ideal patient care unit from a research utilization perspective from our analyses, it is important to note that we did not collect data on several potentially important contextual factors. For example, Greenhalgh *et al*. [107], in a review of the diffusion of service innovations, identified several structural factors that have been shown to influence the likelihood of innovation adoption (*e.g*., size, bed capacity, functional differentiation, decision-making structure, slack resources). Future research examining research utilization patterns at the unit level should incorporate such structural factors.

Aggregating individual nurse scores on a variable of interest to obtain scores for the unit on that characteristic can also introduce bias into the findings if the variable takes on a different meaning and thus has different effects at various levels of analysis. Reliability and validity measures for the following variables of interest raise questions about their suitability for aggregation: attitude, critical thinking dispositions, workload (influence of students), and some unit culture variables (*e.g*., importance of access to continuing education, work values – creativity, work values – efficiency, questioning behavior).

Finally, we adjusted the research utilization score used in the correspondence analysis by taking a weighted average of the score obtained from asking the question on three separate occasions throughout the survey. We assigned higher weights to the research utilization question each time it appeared in the questionnaire because we hypothesized participants learned more about research utilization over the course of questionnaire completion. However, it is also possible that participants may have obtained higher scores on the question each subsequent time it appeared because they learned how to answer the question.

## Conclusion

Our findings offer preliminary support for the argument that context matters. Contextual factors at the patient care unit level, in addition to individual nurse characteristics, were important to promoting research utilization by nurses. By studying several different patient care units, we were able to suggest modifiable components of context at the patient care unit level that may be important determinants of nurses' use of research. We were also able to model an archetypical patient care unit, that is, a patient care unit displaying features optimal for research use. Contextual features identified for such a unit included: higher reported unit culture [as measured by importance of access to continuing education, work values (creativity and efficiency), questioning behavior, and co-worker support] and lower reported environmental complexity (as measured by changing patient acuity and re-sequencing of work). These factors represent modifiable conditions in the hospital environment and have important practical implications for work and unit structures and for organizing nursing service delivery to enhance nurses' use of research findings to improve patient outcomes.

## Competing interests

The authors declare that they have no competing interests.

## Authors' contributions

CAE conceived the study and its design, secured funding, provided leadership and coordination for the two projects and participated in data analysis and interpretation, writing, and final approval of the submitted manuscript, SS, KMcG, and JPM participated in data collection and concurrent data analysis, SS participated in drafting the manuscript, JES made substantial contributions to data analysis and interpretation and made major contributions to writing of the manuscript, BS participated in conceptualization of study, securing grant funding, in the start-up of the study (with data collection) and served as a lead investigator for the pediatric study, coordinating one of the participating sites, JWW participated in conceptualization of study, securing grant funding, in the start-up of the study (with data collection) and served as a lead investigator for the adult study, coordinating one of the participating sites, JL participated in conceptualization of study, securing grant funding and in the start-up of the study (with data collection), LOP participated in study conception, served as a senior advising member on work environment measures, and funded the collection of workload data in two hospitals. KGB participated in interpretation of the findings, GD participated in study conception, data collection and interpretation, GB participated in start-up of the study helping to shape the sampling and data collection activities, CKH coordinated the data linkage activities, participated in data analysis and interpretation, and provided critical commentary, JW participated in data analysis and interpretation, provided critical commentary and served as senior advisor to the team and principal investigator. All authors read and approved the final manuscript.

## Supplementary Material

Additional file 1 additional table 1. Instrument Properties. Click here for file

Additional file 2 additional table 2. Number of Nurses Participating by Unit Click here for file
