# Supplementary material for: Patterns of research utilization on patient care units
Source: Implement Sci. 2008 Jun 2;3:31. doi: 10.1186/1748-5908-3-31 (PMC2490687; doi:10.1186/1748-5908-3-31)
Supplement: Additional file 1 — additional table 1. Instrument Properties. [file 1748-5908-3-31-S1.doc]

Additional File 1. Instrument Properties

| **Instrument** | **Variables Chosen** | **Rogers’**  **Theory** | **Sample Survey Question(s)/Components** | **Item(s)** | **Scale** |
| --- | --- | --- | --- | --- | --- |
| DEM | Gender | Adopter | Gender | 1 | 1-Famale  2-Male |
|  | Age | Adopter | The year that you were born | 1 |  |
|  | Education | Adopter | What is your highest completed level of formal nursing education | 1 | 1-LPN  2-RN diploma  3-Bachelor’s Degree  4-Master’s Degree  5-PhD |
|  | Years in nursing | Adopter | Approximately how many years you have worked in nursing? | 1 | Years |
|  | Usual shift length | Adopter | What is your usual shift length? | 1 | Hours |
| RU | Overall research utilization [[1]](#footnote-2) | Adopter | Overall, in the past year, how often have you used research in some aspect of your nursing practice | 1 | 7-point Likert scale  (never to nearly every shift) |
|  | Authority to use research | Adopter | Avoided using research in direct way because you did not believe you had the authority to do so, even though you were convinced of the usefulness of the research | 1 | 1-5 Likert scale  (never to always) |
|  | Attitude toward research | Adopter | Research makes positive difference to patient care and outcomes | 1 | 1-5 Likert scale  (strongly disagree to strongly agree) |
|  | Intent | Adopter | Would you use research more often in your practice if you could? | 1 | 0-No  1-Maybe  2-Yes |
|  | Belief suspension | Adopter | How often do you actually implement research when it contradicts something you…   1. Learned prior to nursing school 2. Learned in nursing school 3. Learned in your place of work | 3 | Mean scores of 3 items  (Cronbach’s α=.85)  1-5 Likert scales  (never to often) |
|  | People support | Environment | Degree to which the following people are supportive of you using research in your practice:   1. Other nurses in your area 2. Your immediate supervisor 3. Nursing administration 4. General administration 5. Physicians 6. Other health professionals | 6 | Sum of the six items  (Cronbach’s α=.89)  1-5 Likert scales  (not at all supportive to very supportive) |
|  | Organizational support | Environment | To what extent are the following organizational factors present in your workplace?   1. Nurses/others with research skills 2. Paid time allotted for participation in various research activities 3. Attendance at research and clinical conferences encouraged 4. A group or committee to review and critique research 5. Money from internal and /or external sources for research | 5 | Sum of the five items  (Cronbach’s α=.85)  1-5 Llikert scales  (not at all to always) |
| ECS | Re-sequencing of work | Environment | Please rate how each item influenced your ability to provide care required for patient(s) on this shift:   1. Rushing to complete work 2. Doctors not answering pages 3. Completing other’s work 4. Interruptions 5. Multiply delays | 5 | Sum of the five items  (Cronbach’s α=.81)  Scales were coded 0-10 (high increase to high decrease) |
|  | Influence of students | Environment | Please rate how each item influenced your ability to provide care required for patient(s) on this shift:   1. Students on the unit today required supervision and assistance 2. Students wanted access to charts, equipment and supplies | 2 | Sum of the two items  (Cronbach’s α=.75)  Scales were coded 0-10 (high increase to high decrease) |
|  | Changing patient acuity | Environment | Please rate how each item influenced your ability to provide care required for patient(s) on this shift:   1. Patient agitated, confused, restless 2. Unanticipated increase in patient acuity 3. Stat blood work 4. Language barrier with patient/family 5. Extra vital signs 6. Extra charting and paperwork 7. Greater demand for routine teaching 8. Greater demand for psychosocial support for patient 9. Greater demand for psychosocial support for family | 9 | Sum of the nine items  (Cronbach’s α=.84)  Scales were coded 0-10 (high increase to high decrease) |
| NUCAT3 | Co-work support | Environment | My group’s typical behaviour:   1. How important is it to care for your co-workers? 2. How important is it to provide emotional support for your co-workers? | 50 items in total NUCAT3 | Sum of the two items  (Cronbach’s α=.72)  5-point Likert scale  (not at all to extremely) |
|  | Questioning behaviour | Environment | My group’s typical behaviour:  How acceptable is it to question a physician’s order? |  | 5-point Likert scale  (not at all to extremely) |
|  | Continuing education | Environment | My group’s typical behaviour:   1. How important is it to attend in-service classes? 2. How important is professional growth and development? 3. How important is it to attend university classes for a degree? 4. How important is it to learn new technologies? |  | Sum of the four items  (Cronbach’s α=.64)  5-point Likert scale  (not at all to extremely) |
|  | Work values:  creativity | Environment | My group’s typical behaviour:  How important is it to be creative in the nursing care you give? |  | 5-point Likert scale  (not at all to extremely) |
|  | Work values:  efficiency | Environment | My group’s typical behaviour:  How important is it to work in an efficient manner? |  | 5-point Likert scale  (not at all to extremely) |
| PRN80 | Respiratory | Environment | Respiratory category |  | Patient related needs based on frequency and complexity |
|  | Feeding & hydration | Environment | Feeding & hydration category |  |  |
|  | Elimination | Environment | Elimination category |  |  |
|  | Hygiene | Environment | Hygiene category |  |  |
|  | Communication | Environment | Communication category |  |  |
|  | Treatments | Environment | Treatments category |  |  |
|  | Diagnostic procedures | Environment | Diagnostic procedures category |  |  |
|  | PRN80Total | Environment | All PRN80 items |  | Sum the above seven categories and multiply by 5 minutes |
| CCTDI | Truth-seeking | Individual | Seek the truth; courageous about asking questions; honest and objective about pursuing inquiry | 12 | Cronbach’s α coefficients are.52 - .74  Each item with a 6-point Likert scale (strongly disagree to strongly agree ) |
|  | Open-mindedness | Individual | Tolerant of divergent views: sensitive to the possibility of one’s own biases; respect the right of others to hold different opinions | 12 |  |
|  | Inquisitiveness | Individual | Have intellectual curiosity; value being informed; eager to know how things work; value learning for learning’s sake | 10 |  |
|  | Systematicity | Individual | Organized; focused; diligent in inquiry | 11 |  |
|  | Maturity | Individual | Reflective in own judgments; possess cognitive maturity; strive for epistemic development | 10 |  |
|  | Self-confidence | Individual | Trust in own reasoning processes | 9 |  |
|  | Analyticity | Individual | Alert to potentially problematic situations; anticipate possible results or consequences; prize the application of reason; use of evidence | 11 |  |
|  | CT total |  | All CCTDI items | 75 | Sum of the 75 items |

1. Definition of overall research utilization - The use of any kind of research findings (nursing and non-nursing), in any kind of way, in any aspect of your work as a registered nurse. [↑](#footnote-ref-2)
