# Supplementary material for: Patterns of research utilization on patient care units
Source: Implement Sci. 2008 Jun 2;3:31. doi: 10.1186/1748-5908-3-31 (PMC2490687; doi:10.1186/1748-5908-3-31)
Supplement: Additional file 2 — additional table 2. Number of Nurses Participating by Unit [file 1748-5908-3-31-S2.doc]

.**Additional File 2. Number of Nurses Participating by Unit**

| **Adult/pediatric** | **Unit Code** | **Data Collection** | |
| --- | --- | --- | --- |
|  |  | **Time 1** | **Time 2** |
| **Adult** | Unit 1 | 29 | 26 |
|  | Unit 2 | 34 | 25 |
| **Pediatric** | Unit 3 | 13 | 7 |
|  | Unit 4 | 19 | 7 |
|  | Unit 5 | 14 | 11 |
|  | Unit 6 | 49 | 29 |
|  | Unit 7 | 18 | 12 |
| **Total:** |  | 176 | 117 |
